# Balancing obligations: should written information about life-sustaining treatment be neutral?

**DOI:** 10.1136/medethics-2013-101965

**Published:** 2014-04-24

**Authors:** Vicki Xafis, Dominic Wilkinson, Lynn Gillam, Jane Sullivan

**Affiliations:** 1 Perinatal Ethics Unit, Discipline of Obstetrics and Gynaecology, Robinson Institute, The University of Adelaide, North Adelaide, South Australia, Australia; 2 Department of Neonatal Medicine, Robinson Institute, The University of Adelaide, Adelaide, South Australia, Australia; 3 Children's Bioethics Centre, The Royal Children's Hospital, Melbourne, Victoria, Australia; 4 Centre for Health and Society, University of Melbourne, Children's Bioethics Centre, The Royal Children's Hospital, Melbourne, Victoria, Australia

**Keywords:** End of Life Care, Clinical Ethics

## Abstract

Parents who are facing decisions about life-sustaining treatment for their seriously ill or dying child are supported by their child's doctors and nurses. They also frequently seek other information sources to help them deal with the medical and ethical questions that arise. This might include written or web-based information. As part of a project involving the development of such a resource to support parents facing difficult decisions, some ethical questions emerged. Should this information be presented in a strictly neutral fashion? Is it problematic if narratives, arguments or perspectives appear to favour stopping over continuing life-sustaining treatment? Similar questions might arise with written materials about decisions for adults, or for other ethically contentious decisions. This paper explores the meaning of ‘balance’ in information provision, focusing particularly on written information about life-sustaining treatment for children. We contrast the norm of non-directiveness in genetic counselling with the shared decision-making model often endorsed in end-of-life care. We review evidence that parents do not find neutrality from medical professionals helpful in discussions. We argue that balance in written information must be understood in the light of the aim of the document, the most common situation in which it will be used, and any existing biases. We conclude with four important strategies for ensuring that non-neutral information is nevertheless ethically appropriate.

## Background

Decisions about the provision or not of life-prolonging treatment are potentially complex, fraught with uncertainty, and deeply personally challenging. This may be especially so when the patient is a child and their parents are making decisions on their behalf. Parents may struggle to decide whether they should continue to provide treatments or whether they would be helping their child suffer less by limiting or stopping treatment. Health professionals are an important source of information and support and help to clarify which options are best for each child, but parents frequently seek other sources to help them deal with the medical and ethical questions that arise.^1–8^ Increasingly, parents turn to the internet for information or support.^2^ ^4^ ^5^ ^8^ ^9–13^ However, there is relatively little available material for parents about important considerations in life-sustaining treatment decisions. For example, there is sometimes disagreement between healthcare professionals about the life support treatment that is being provided to a child. Written information about how such disagreements arise and what parents can do in such situations is non-existent.

In recognition of the paucity of reliable written resources that assist in clarifying complex medical and ethical issues relating to parental decision-making for their seriously ill or dying child, we recently developed a booklet and comprehensive on-line resource, titled ‘Caring Decisions’, to support parents facing such difficult decisions.^14^

Our handbook is written in question and answer format, and addresses a wide range of questions that parents might ask or contemplate. Primarily, these resources tackle the difficult questions surrounding stopping or not providing treatment and place these in the context of ethically and medically appropriate and caring decisions for the child. The handbook is designed to support parents within a shared decision-making model, wherein parents and healthcare professionals make decisions together about life-sustaining treatment for a child. This is the prevailing model in Australia, as in many parts of the world.^15^ In the course of preparing this material and during an extensive consultation phase with health professionals, support groups, which included parents whose children had been in life-threatening situations (such as the Miracle Babies Foundation, Bliss, and Very Special Kids), and bereaved parents, we encountered the following ethical question: must such material be presented in a strictly neutral fashion?^i^

On review of our material, it was clear that issues and arguments in favour of stopping or not providing treatment had been given more space and attention than issues in favour of continuing or starting life support (box 1). We also included quotations and stories from parents who had faced life-sustaining treatment decisions for their child. For example: “We were told there was no cure but he could be kept alive longer … but it would not be a way that would be comfortable for [our child], and it would be very invasive … So we just decided, we wanted to do it the gentle way … And hope that we can do that for as long as possible”. However, these quotations are largely from parents who have been involved in end-of-life decisions, and provide support for decisions to limit or discontinue treatment.

Box 1 **Excerpts from written information for parents about end-of-life decisions**

Caring Decisions, Australia (non-neutral)^14^

“*If I agree to stop life support, will people think I am a bad parent?”*

“If the doctors are talking to you about life support for your child, it is because they are worried that continuing life support treatment would not be helpful.

Making a decision to stop life support in these circumstances is not a sign of being a bad parent. On the contrary, it can be the most caring, loving thing a parent can do for their child.”

“Is stopping life-support treatment giving up on my child?”

“Sometimes families feel like they would be ‘giving up’, or letting their child down if they agree to stop life support treatment.

As parents, you will never give up on your child. Your love for them will never stop. But you may need to give up on medicine or on medical treatments that are not going to work. Doctors cannot treat every sickness. Although we find it easier to accept the death of older people, sometimes children get sick too, and doctors cannot save them.”

Hard Choices for Loving People, USA (non-neutral)^16^

“Children with multiple organ system failure or those in the terminal phase of a disease have little chance of surviving CPR. What makes the decision to withhold resuscitation attempts on these little ones so difficult is the overwhelming sense of loss for the parents and for the medical staff. For a parent to say ‘do not resuscitate’ symbolizes the lost future of the child and lost hopes of the parents. The physician and other healthcare workers can help sort out the ‘medical side’ of this decision. The more difficult part is letting go.” (p. 15)

Life's Toughest Moments, USA (non-neutral)^17^

“Your healthcare team may have talked with you about your child's health status clearly not improving. They may have been, in essence, attempting to revive your child for hours, days, maybe even weeks or months. Although the process of trying to cure your child has gradually led to more and more treatments, CPR is the last possible option. There comes a time when curative treatment must shift to comfort treatment.” (p. 3)

Making Critical Care Decisions for Your Baby, Bliss, UK (neutral)^18^

“It might help you to know more about some of the care and treatment options for a very sick baby. The next section of this booklet explains what happens with each of these possibilities:

Continuing intensive care

Stopping intensive care

Moving to palliative care

Whichever option you and your baby's care team choose, the doctors and nurses will always work to keep your baby comfortable, relieve any pain and support you as a family.” (pp. 8–11)

While developing the ‘Caring Decisions’ handbook, we systematically searched the published and grey literature for written material to support parents facing end-of life decisions. Excerpts from some of these are included in box 1. Some of this material appears, like our own, to provide more support for withdrawing or withholding treatment, while other material is more neutrally written. Which is the better approach? Is it ethically problematic if narratives, arguments or perspectives appear to favour stopping rather than continuing life-sustaining treatment? In answering this question, we will focus on balance in written materials. However, some of this analysis is also relevant to verbal interactions between parents and healthcare professionals.

## Neutrality in information provision

It is helpful to have a clear definition of what we understand as being neutral information provision/counselling. One way of defining this is:

Neutral information provision: The presentation of opposing alternatives or points of view without communicating a preference or recommendation for any option presented.

Favouring one or the other alternative could be communicated overtly or it could be communicated more subtly by giving greater prominence to one of the alternatives either in space or time. Neutrality, therefore, appears to mandate giving equal weight to different options. In addition to equal weight given to different options presented, neutrality may also be expressed via the choice of words and phrases used either verbally or in written materials.^ii^

Outside the medical context, when media outlets present information in an election campaign, for example, they are encouraged to remain neutral to the political parties and avoid direct or implicit favouring of any individual party. A number of Western democracies have varying levels of regulation or statutes that require the provision of equal airtime to political parties.^19–21^ Such an approach in a democratic political context appears to be entirely appropriate.

Neutrality is sometimes endorsed for counselling about morally controversial medical decisions. In particular, genetic counselling following antenatal diagnosis is often encouraged to have a non-directive and value-neutral approach.^22^ While there is no single accepted definition of non-directiveness in genetic counselling,^23^ ^24^ it seems to be generally agreed that it involves the communication of all relevant information without the provision of advice or personal recommendations.^25^ Support is provided to aid patients to make the best decision from their own perspective, but the counsellor does not guide them towards a particular decision.^26^ ^27^ Although non-directiveness has been criticised as not being feasible^22^ ^27^ ^28^ or useful,^29^ it still forms a key part of professional guidelines relating to genetic counselling.^30^ ^31^

There is a range of reasons why neutrality is encouraged in genetic counselling. These include the importance of patient autonomy, the controversial and contested nature of decisions about termination of pregnancy, and a perceived need to distance medical professionals from concerns about eugenics.^27^ Patient/parent decisions are closely linked to personal, religious, cultural and ethical values that may not be shared with health professionals. Although non-directiveness usually relates to counselling of patients, it has also been applied to written information about genetic counselling and prenatal decisions.^32^

Similar sorts of considerations might be considered to be at stake for decisions regarding life-sustaining treatment for children, and so it might be thought that counselling or written material in this setting should also be neutral. On the surface, neutrality of information may appear to promote autonomous decision-making and be regarded as a morally appropriate approach to adopt both when speaking to parents and when preparing written materials for parents faced with such difficult decisions. For example, the booklet ‘Making critical care decisions for your baby’ produced by the charity Bliss in the UK, adopts such a neutral approach (box 1). The booklet devotes equal space (half a page) to the three different options of *Continuing intensive care*, *Stopping intensive care* and *Moving to palliative care* (pp8–11) and does not appear to favour any of them. Interviews with a small group of Canadian neonatologists revealed that they sought to be objective and neutral when counselling parents prior to delivery of an extremely premature newborn infant. “I give them the most neutral information possible so that they could make the decision whether they want their child to be given care or not (p. 1492)”. 33

Is a neutral approach the right one for information provision and counselling relating to life-sustaining treatment decisions? While parents find certain verbal and non-verbal behaviours from healthcare professionals coercive and distressing,^5^ qualitative research with parents who have experienced the death of their child suggests that neutrality or non-directiveness may not always be seen as appropriate or desirable.^33^ In this large study interviewing parents whose child had died in neonatal intensive care, those parents who perceived that they had been left to decide about treatment for their seriously ill child without input from healthcare professionals later struggled with the moral acceptability of the decision they had made.^33^ In a Canadian study, parents reported that neutral provision of information without any other involvement from healthcare professionals was unhelpful; health professionals were reported to have delivered the scientific information and left, without developing any form of relationship, thus leaving parents unaided in the decision-making process.^34^ In another study, Keenan and colleagues found that directiveness in counselling about resuscitation of extremely premature infants was not perceived as detrimental by parents.^35^ Interestingly, this study also found a discrepancy in perceptions of directiveness between genetic counsellors and mothers, with 67.7% of mothers indicating that the counsellor had made a treatment recommendation and only 27% of counsellors believing that they had made a recommendation.^35^

## Balance in information provision

If a neutral approach to information provision is not necessarily the right one, what are the alternatives? The concept of ‘balance’ may prove helpful:

Balanced information provision: The presentation of opposing alternatives or points of view with unbiased weight given to different options.

Balanced counselling or information provision might be appropriately neutral in some circumstances. However, where there is greater evidence or there are stronger normative reasons in favour of one option, a balanced approach will not be neutral between options. Correspondingly, in such situations a neutral approach would be decidedly unbalanced. So in defining ‘balanced’ information provision, the key is that any weight given to a particular option should be ‘unbiased’, in the sense that it is ethically justified weight.

While election coverage might be appropriately neutral, for other issues in public discourse a balanced approach would be preferable. For example, news services like the BBC have been criticised for attempting to remain neutral in their presentation of issues around climate change or immunisation scares.^36^ A strictly neutral approach appears to lead to highly unbalanced presentation of issues and would seem to require giving, for example, equal weight to astrologers and to astrophysicists when discussing the movement of planets. When there is strong and clear scientific evidence in favour of one option or alternative, it is not credible to present these options/alternatives neutrally. The same would potentially apply where there are clearly stronger ethical arguments in favour of one option. To illustrate, a balanced but non-neutral approach would be more appropriate when presenting information about child-sex-slavery or female genital mutilation. Is a balanced, as opposed to a neutral, approach desirable for counselling about life-sustaining decisions? If there are some situations in which it is clear that withdrawing or withholding of treatment is the ethically more appropriate decision, then in these situations, balance rather than neutrality is required.

‘Balanced information provision’ gives unequal weight to options where there is stronger scientific evidence or stronger normative reasons in favour of a particular alternative. Although evidence and argument may both count in favour of limitation of life support in some circumstances (eg, in a child dying in intensive care despite maximal support), they would count against limitation of life support in other circumstances (eg, where a child is likely to survive with a good quality of life if treatment is provided). How would unequal weight be justified in written material where a specific child's situation is not known?

## In favour of the ‘balanced’ approach for counselling about life-sustaining treatment

The most widely endorsed approach in counselling and decision-making involving parents of seriously ill or dying children is that of ‘shared decision-making’.^37–40^ There are variations to the shared decision-making model but, in general terms, it involves parents to varying degrees with decisions made in accordance both with their preferred style of decision-making^41^ and the context within which the decision is being made.^33^ While researchers may develop clear-cut categories regarding decision-making models,^42^ parents themselves often think of shared decision-making in different ways.^1^ The spectrum of parental involvement ranges from parent involvement in discussions but end-of-life decisions made by medical professionals,^41^ through consensus decision-making by parents and health professionals together,^42^ to final decisions being made largely or entirely by parents^43^ with professional support.

There are several reasons why the shared decision-making model is appropriate for life-sustaining treatment decisions in children. Importantly, this approach eschews strict neutrality in information provision and counselling. Decisions require input from healthcare professionals as they have the medical expertise required to evaluate a child's likely prognosis and response to treatment. However, it is parents who have in-depth knowledge regarding what is best for *their* child, they are most affected by any decision made, and they know what course of action best fits with their values and beliefs or, when the child is older, their child's preferences. When values and beliefs are taken into account during the shared decision-making process, parents have reported less grief after the loss of their child.^42^ Parental involvement in discussions can prove extremely important in uncovering aspects relating to the child's history, personality, condition or treatment that might otherwise not have been discussed and which might be central to subsequent treatment decisions. Despite parents’ desire to be involved in decision-making,^33^ ^42^ ^44–51^ some parents report that having full responsibility for decisions relating to withdrawing or withholding treatment is an unbearable weight to bear alone; therefore, sharing the burden of such decisions with medical professionals is welcomed by parents.^33^ ^41^ ^42^ ^44^ ^52–54^ Even if parents take ultimate responsibility for the decision, it is still important for them to have information about what the doctors believe is the ethically appropriate decision. Provided this information is given sensitively and with clear explanation of the reasons, it need not be at all coercive. Arguably such information is a crucial part of the set of information that is material to a parent's decision, and therefore necessary if the parent's decision is to be an autonomous, well-informed one. This is why it is important for healthcare professionals not to remain neutral in the provision of medical facts but to signal which option is in the child's best interest taking into account all the relevant facts.

Shared decision-making necessarily supports a balanced approach to counselling, with health professionals providing advice or endorsement of options in accordance with parents’ values and the professionals’ assessment of the child's best interests.^42^ One challenge, however, is whether this model of decision-making can be applied to written information. Such information is likely to be accessed by parents in a wide range of different situations. It is necessarily general in nature and cannot address the specific circumstances of particular children, nor can it be ‘interrogated’ for clarification to ascertain whether specific information relates to the specific circumstances of the persons reading it. It is also not possible to check the readers’ understanding of the material. In addition, written material cannot take into account parents’ preferred decision-making style and the level of support that they want or need.^iii^ The on-line resource is slightly better placed to do so because it allows somewhat more scope for parents to seek out information specifically relevant to their situation. However, the wording of any section they choose to read is fixed and framed in a general rather than context-specific way. It is somewhat difficult, then, to see written information as contributing to a form of ‘shared decision-making’.

One solution would therefore be for written material to be used as an adjunct to counselling by health professionals. ‘Caring Decisions’ emphasises that parents should discuss material that they have read with their child's doctors. It explicitly acknowledges the importance of shared decision-making. However, the question remains: if written material is going to be used in a variety of situations, and in conjunction with counselling, should it not be neutral?

A separate reason for providing more balanced material relates to the context in which decisions are made. The healthcare system is designed to preserve life and intently focuses on this aim (particularly in children). This introduces a bias towards the preservation of life that is deeply engrained both in Western thinking and in our healthcare systems. Parents rarely, if ever, have to specifically consent to providing intensive care or life support. In fact, in some contexts, there is such a great desire to preserve life from the healthcare professionals’ perspective that parents’ reasonable requests that life-sustaining treatment for their child be withdrawn are sometimes ignored or denied.^55^ Secondly, when faced with life-sustaining treatment decisions, most parents instinctively want everything possible done to preserve their child's life^2^ ^35^ ^43^ ^56^ and often initially consider this to be the only acceptable option. Parents’ strong and instinctive desire to preserve their child's life plays a significant role in the way they think about the course of action for their child. In essence, when we approach life-sustaining treatment decisions that parents have to make for their child, the scales are not evenly balanced to start with but are heavily skewed in favour of continuing or providing the child with treatment.^iv^

Awareness of the context for decisions, and the psychological and social pressures that parents as decision-makers face, was one of the motivations behind producing the parent handbook. It is likely that this also influenced the writing of other authors’ material relating to life-sustaining treatment decisions. In general, parents or surrogates do not need support or help to make a decision to provide cardiopulmonary resuscitation, or intensive care, or continued medical therapy. The difficult decision for parents is the one to limit or withdraw treatment. The aim of our handbook was to openly and honestly discuss issues of withdrawal or limitation of treatment, and also to help families to come to terms with and to accept that such decisions were not merely alternatives to consider but could be the most caring decisions.

## Concerns about non-neutrality

There are, however, arguments against non-neutral provision of information about life-sustaining treatment decisions.

### Inappropriate withdrawal/limitation of treatment

One potential concern is that written information apparently favouring withdrawal/withholding of treatment might influence parents and lead them to decide to stop or withhold treatment for their child when such a decision would be contrary to the child's best interests. For example, parents might not wish treatment because of concern about a newborn infant's future quality of life even though the child is likely to be only mildly impaired.^57^ This concern is unfounded, however, as it is inconceivable that decisions would be made in isolation from discussions with healthcare professionals. It also seems highly unlikely that healthcare professionals would agree to limitations of treatment that were not in a child's best interests. Professional guidance and legal precedents do not permit parents to decline clearly beneficial treatment.^37^ ^58^ Given the strong legal, medical and social norms in favour of preservation of life, if there were a difference of opinion, treatment would almost certainly be provided or continued.

### Coercion/autonomy

A second objection might be that written materials that favour non-treatment would be coercive or would undermine parents’ autonomy to decide for their child. It is important to be clear about what we mean by coercion, and what we take to be autonomous decision-making. Coercion typically involves force, threats or undue influence^59^ designed to lead to a particular decision by someone in a less powerful position who typically stands to suffer if they do not comply. Not all influences on decisions are coercive.^59^ ^60^ Autonomous decisions do not necessarily need to occur in a vacuum, independent of any external influence^59^; indeed, if we take this to be the standard of autonomous decision-making, none of our decisions are truly autonomous. Parents who find themselves facing life-sustaining treatment decisions for their child may be vulnerable in many ways, including emotionally, psychologically, physically, socially and cognitively. However, the sensitive and compassionate articulation of difficult medical and ethical issues cannot reasonably be considered to be coercive.

## Balancing obligations

We have argued in favour of adopting a balanced approach to the provision of written information for medical decisions. We have focused on life-sustaining treatment decisions in children, and argued that in this situation, written information does not need to be strictly neutral (and that there is benefit in non-neutrality). Similar considerations are likely to apply to other ethically contentious decisions.

However, where this approach is taken we propose three important strategies for achieving the right balance, and for ensuring that non-neutral information is nevertheless ethically appropriate.

1. First, written information should be provided as a supplement to appropriate counselling. Written material should make clear that the information provided is of a general nature and may or may not apply to specific cases. In ‘Caring Decisions’ we strongly encourage parents to discuss issues raised in the publication with their child's healthcare professionals.
2. Second, it is vitally important that written material addresses and explicitly acknowledges all options that could be appropriate. This means providing at least some weight to contrasting points of view and perspectives. For life-sustaining treatment decisions, even if greater emphasis is placed on decisions to withdraw or withhold treatment, it is important to clearly acknowledge that continuation or provision of treatment is sometimes both acceptable and appropriate.
3. Third, where material gives additional emphasis to some options, this should be acknowledged explicitly, and justified.^v^
4. Finally, any such written information should be exposed to rigorous consultation and review processes involving patients/families and health professionals, as well as community agencies. If parents who have faced end-of-life decisions find the relative weight given to different options appropriate, we have strong presumptive evidence that the material is, in fact, balanced.

## Footnotes

i We have underway a separate study that asks parents whether the material in the resources was helpful, and asks professionals about the impact of the resources on decision-making, including its influence on conflicts.

ii In verbal exchanges, paralinguistic features such as voice quality, rate of speech, pitch and tone, as well as rhythm, intonation and stress, all provide nuanced meaning and are important features which reveal the speaker's attitudes and emotions. These features can therefore also communicate neutrality, or absence of, in verbal communication.

iii Technology may overcome this limitation to some degree. For example, it may be possible to tailor on-line medical information to patient's informational needs at that specific moment as well as to their values. The on-line version of ‘Caring Decisions’ gives parents the option to self direct to particular questions or topics that are concerning them.

iv One possibility is that in other cultures the context for end-of-life decision-making may lead to different conclusions. For example, if a culture already had a balanced perspective on end-of-life decisions, there would be no need for written material to give extra weight to withdrawing/withholding treatment. If a culture had an existing bias against continuing life support (for example in the setting of disability), balance would potentially require written information to emphasise the arguments in favour of continuing treatment. (We are grateful to an anonymous reviewer for this point.)

v We are grateful to an anonymous reviewer for this point. In the introduction to ‘Caring Decisions’ we note: “We have tried to provide a balanced perspective on end of life decisions, recognising that in some situations there may not be a single right answer and that it may be appropriate either to provide active life support, or to focus on comfort care. However, we are also aware that for many families the difficult decision, the thing that they find hardest, is to stop (or not start) life support. We have therefore placed a special emphasis on the reasons why it is appropriate and ethical to stop or avoid some treatments when a child is nearing the end of their life, and provide reassurance for families that these decisions are common”.
